# Elevated ozone and carbon dioxide affects the composition of volatile organic compounds emitted by *Vicia faba* (L.) and visitation by European orchard bee (*Osmia cornuta*)

**DOI:** 10.1371/journal.pone.0283480

**Published:** 2023-04-26

**Authors:** Mark Otieno, Zsolt Karpati, Marcell K. Peters, Laura Duque, Thomas Schmitt, Ingolf Steffan-Dewenter

**Affiliations:** 1 Department of Animal Ecology and Tropical Biology, Biocenter, University of Würzburg, Würzburg, Germany; 2 Department of Water and Agricultural Resource Management, University of Embu, Embu, Kenya; 3 Department of Chemical Ecology, Plant Protection Institute, Centre of Agricultural Research, ELKH, Budapest, Hungary; National Research Council of Italy (CNR), ITALY

## Abstract

Recent studies link increased ozone (O_3_) and carbon dioxide (CO_2_) levels to alteration of plant performance and plant-herbivore interactions, but their interactive effects on plant-pollinator interactions are little understood. Extra floral nectaries (EFNs) are essential organs used by some plants for stimulating defense against herbivory and for the attraction of insect pollinators, e.g., bees. The factors driving the interactions between bees and plants regarding the visitation of bees to EFNs are poorly understood, especially in the face of global change driven by greenhouse gases. Here, we experimentally tested whether elevated levels of O_3_ and CO_2_ individually and interactively alter the emission of Volatile Organic Compound (VOC) profiles in the field bean plant (*Vicia faba*, L., Fabaceae), EFN nectar production and EFN visitation by the European orchard bee (*Osmia cornuta*, Latreille, Megachilidae). Our results showed that O_3_ alone had significant negative effects on the blends of VOCs emitted while the treatment with elevated CO_2_ alone did not differ from the control. Furthermore, as with O_3_ alone, the mixture of O_3_ and CO_2_ also had a significant difference in the VOCs’ profile. O_3_ exposure was also linked to reduced nectar volume and had a negative impact on EFN visitation by bees. Increased CO_2_ level, on the other hand, had a positive impact on bee visits. Our results add to the knowledge of the interactive effects of O_3_ and CO_2_ on plant volatiles emitted by *Vicia faba* and bee responses. As greenhouse gas levels continue to rise globally, it is important to take these findings into consideration to better prepare for changes in plant-insect interactions.

## Introduction

Global climate change is triggered by increasing carbon dioxide (CO_2_) concentrations paralleled by higher ozone (O_3_) concentrations, which have direct impacts on plant physiology [1,2] and indirect effects on interactions with associated insects [3]. Currently, such plant-mediated effects on plant-insect interactions have been increasingly clarified but combined effects of O_3_ and CO_2_ are still unknown, in particular for plant-pollinator interactions [4]. Both O_3_ and CO_2_ can disrupt plant-insect communication by changing the Volatile Organic Compounds (VOCs) profile emitted by the plants or by altering nectar quality and quantity [5,6]. Tropospheric O_3_ can affect VOCs by altering plant allocation and inducing changes in VOCs’ emission or by chemically reacting with the emitted VOCs to alter their chemical structure [7]. Some studies have linked elevated ozone levels to reduced photosynthesis as a result of lower performance of stomatal conductance [8,9] and reduced reproductive capacity of plants, foliar damage, increased abscission rates and senescence [2]. However, many papers also indicate that the effect on photosynthesis is through effects on Rubisco rather than stomatal conductance [e.g. 10–12]. Intuitively, altered physiology will affect the normal functioning of the plant, and this may include changed emissions of VOCs. In this case, O_3_ could have indirect effects on VOC emission via modification of the plants’ physiology. Such a modification may, in turn, lead to changes in the concentration or composition of floral scents. When this happens, the visitation could be reduced because the attractiveness of VOCs detected by the pollinators become lower both in quality and in quantity [13]. Studies that simultaneously investigated the effects of O_3_ on VOC emission and consequences for flower visits are rare [but see 13].

Increased CO_2_ concentration in the air can also directly affect insect-plant interactions via altered VOC emission by changing the total emission rates and VOC composition [5]. Also, compositional changes of the VOCs emitted by the plant can act as a repellent for insects [3]. The changes in VOC composition have been documented to impede plant–plant communication [14,15] and between plants and insects compromising the ability of insects to detect VOC cues [16]. Indirectly, elevated CO_2_ can alter VOC emission via modification of photosynthesis and plant growth. Elevated CO_2_ has been linked to higher photosynthesis rates, which increases the amount of carbohydrates in the plant available for greater starch reserves and growth [17,18]. Good physiological processes are responsible for increased plant growth and performance, which can include elevated emission of VOCs [see 19].

Among the plant organs involved in plant-animal interactions that are sensitive to changes in growth and physiology are extra floral nectaries (EFNs). EFNs are important plant organs that produce nectar as a primary reward for the organisms which defend them against herbivory. The nectar produced by these glandular organs is rich in carbohydrates, lipids, and amino acids and attracts a wide diversity of arthropods [20,21]. EFNs are located on plant parts other than the flowers, typically on stipules, petioles, or leaf bases [22], but can also occur on flower buds [23]. The occurrence of EFNs is widespread in the plant kingdom, recorded in over 3,900 species of plants belonging to 806 genera and 110 families [22].

Legume is recognized as the most common EFN-producing plant family and it has a well-known diversity of insect mutualisms [24–27]. Despite the large diversity of bees visiting legume EFNs, the interactions between these bee species and EFNs are still poorly understood. Bees visit flowers primarily to collect pollen and nectar to provide the protein and carbohydrates they need for themselves and to feed the developing larvae [28,29]. Learning to visit high rewarding plants [30] by utilizing multiple traits, including nectar and floral scent has been evolved in bees [31,32]. This allows the bees to locate and collect food with maximum efficiency [30]. In most legume species, flowers secrete little amounts of nectar not sufficient to meet pollinators’ energy needs [33]. This inadequacy forces the insects to forage on plant species that produce large amounts of nectar or utilize extra floral nectar from legume plants, if available.

Bees, e.g., *Osmia cornuta* (Hymenoptera, Megachilidae), are the primary visitors of flowers and EFNs of many legume plants including field bean (*Vicia faba* L., Fabaceae), a globally important pulse crop often grown as monoculture [34–37]. *Vicia faba* plants produce extra floral nectar primarily for ant attraction to defend itself against herbivores [38], but bees also frequently utilize these organs to obtain nectar as an extra reward in addition to the pollen and nectar they obtain from the flowers [39]. Numerous studies have investigated the effects of O_3_ and CO_2_ enrichment both individually and in interaction on plant growth responses in different free-air concentration-enrichment (FACE) experiments [e.g. 18,40–42]. However, none has studied the effects of the two atmospheric gases on VOC emission, EFN nectar volume and sugar concentration.

Although *Vicia faba’s* growth, development, and reproduction are known to be affected by O_3_ and CO_2_ [1,43,44], studies addressing the effects of these gases on EFN nectar production (volume and sugar concentration) are non-existent. Furthermore, the interactive effects of these gases on insect-EFN mutualisms in *Vicia faba* are unknown.

In this study conducted in a greenhouse environment, we tested whether VOCs, nectar volume and sugar concentration from the EFNs of *V*. *faba* are affected by increased O_3_ and CO_2_ levels both independently and in interaction and whether there are consequences for bee visits to these EFNs. *Vicia faba* was selected as a ubiquitous crop in Europe and a crucial global food security and bioenergy crop with existing knowledge on its reproduction to understand how the likely rise in atmospheric pollution could impact the interactions with its pollinators.

## Materials and methods

This study was conducted through a series of experiments from January to April 2020 in a greenhouse located at the Biocenter, the University of Würzburg in Germany. *Vicia faba* plants were exposed to elevated levels of O_3_ and/or CO_2_. EFN nectar secretion of the plants and the behavior of the European orchard bee (*Osmia cornuta*, Latreille, Megachilidae) were assessed later under normal atmospheric conditions.

### Crop establishment

The *Vicia faba* variety Fuego, a regionally important cultivar in Bavaria, Germany, was used in the experiments. The crop was planted in 18 cm x 18 cm pots filled with a peat-based rooting media composed of a 2:1 ratio of peat (Einheits Erde CL ED 73) and sand (Hamann Filters and 0.7–1.25 mm). Greenhouse environmental conditions were controlled at 25°C in 16 hours of light, and at 18°C in 8 hours of dark.

In the greenhouse, there were two pests noticed during the experiment; the fungus gnats (*Sciara hemerobioides*: Sciaridae) and the western flower thrips (*Frankliniella occidentalis*: Thripidae). The pests were controlled as described by [45]. We used the nematode, *Steinernema feltiae*: Steinernematidae from Katz Biotech AG (www.katzbiotech.de) to control the fungus gnats eggs and larvae. The nematode was added to the soil at a rate of 50 million per 100 m^2^ as recommended by the manufacturer. The Adult insects of this pest species were controlled using yellow sticky traps. Predatory mites *(Amblyseius cucumeris*: Phytoseiidae) from Katz Biotech AG and Biobest^®^ (www.biobestgroup.com) were used to manage the western flower thrips (*Frankliniella occidentalis*: Thripidae) on the plant leaves and flowers at the rate of 50/m^2^.

Two cohorts, each with 32 plants, were planted on the 2^nd^ and 12^th^ January 2020. The plants were watered every three days, and pests were managed using established biological control measures.

### Fumigation experimental set-up

**Fig 1:**
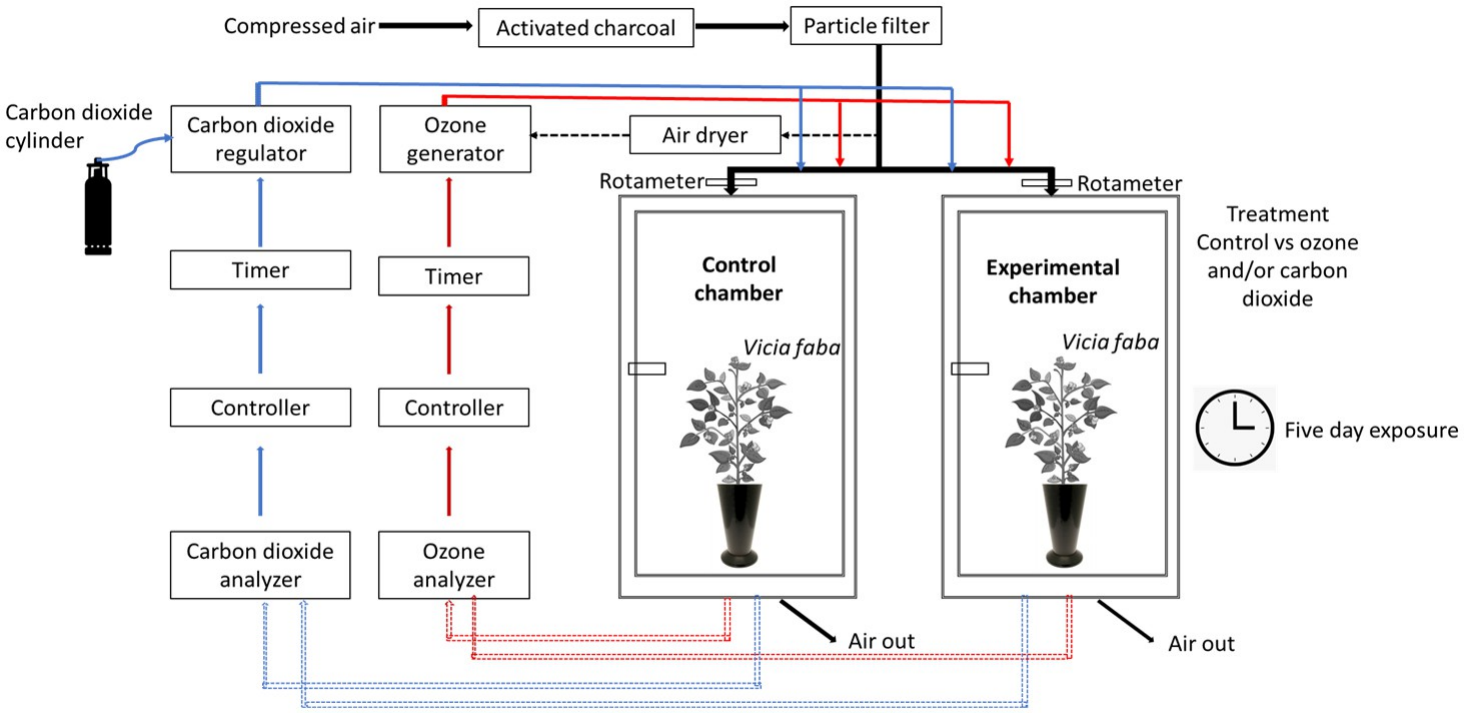
Representation of the ozone—Carbon dioxide exposure system experimental setup.

The fumigation set-up was comprised of components assembled as described in details in [45–47]: The setup (Fig 1) had:

1. Two glass chambers (chamber 1 and 2) made of a stainless-steel door frame each with an approximate volume of 1000 dm^3^;
2. An O_3_ gas generator (INNOTEC high engineering GmbH) linked to an air dryer (AIRdryer3.2, INNOTEC);
3. A CO_2_ cylinder linked to a regulator calibrated to allow for the amount of CO_2_ required in the chamber. The CO_2_ regulator was an assembly comprising seven components manufactured by Grow Control Company (www.growcontrol.de). The assembly had (a) manometer cylinder pressure, (b) flow rate indicator, (c) shut-off valve, (d) Solenoid switching valve, (e) flow adjustment knob, (f) cylinder connector and (g) upper pressure valve;
4. An O_3_ analyzer (APOA-370, Horiba Ltd.);
5. A CO_2_ analyzer (GrowControl GrowBase EC Pro) linked to the regulator described in (iii) above;
6. A controller that links the O_3_ analyzer and the O_3_ generator that was used to regulate the O_3_ concentration in the chamber;
7. A timer;
8. A primary stream of compressed air allowed to pass through an activated charcoal filter and a particle filter before it reaches the chambers;
9. A secondary stream of air branching from the primary one, passing through the air dryer and the O_3_ generator and discharges in the main air stream, allowing for O_3_ enrichment of the incoming air;
10. 2 rotameters that allowed for leveling the amount of incoming air to each chamber to 70 L/min.

The photoperiod conditions were the same as in the greenhouse itself (16 hours of light, and 8 hours of dark from 5 am to 8 pm) because the two chambers were almost exclusively made of transparent glass. The two chambers allowed us to have, at any given time, an O_3_-clean environment and normal CO_2_ levels in one chamber (chamber 2) and an O_3_ and/or CO_2_ enriched environments at different times of the fumigation experiments (chamber 1). The O_3_ and CO_2_ analyzers were constantly sucking in the air from the chamber used for the treatments with these gases and analyzing it for its O_3_ or CO_2_ content or both depending on which fumigation experiment was running. This information was then passed on to the controllers that give feedback to the CO_2_ and O_3_ generators, switching them on or off accordingly in order to achieve the desired average CO_2_ or O_3_ concentrations, which could be modified in the controller.

Fumigation consisted of exposing the plants in the chambers for five continuous hours/day between 5 am and 8 pm for five consecutive days. Sets of eight plants in the stage of flower initiation were exposed to one of the four following treatments:

1. Control—Exposure to air purified with charcoal filters in chamber 2. The O_3_ and CO_2_ concentrations were not permanently monitored in this chamber, but pretesting revealed that the mean concentrations in this chamber were ~0 ppb and ~346 ppm for O_3_ and CO_2_, respectively.
2. O_3_—exposure to enhanced levels of O_3_ (120 ppb).
3. CO_2_—exposure to enhanced levels of CO_2_ (900 ppm).
4. O_3_ + CO_2_—simultaneous exposure to enhanced levels of O_3_ and CO_2_ at the above enhanced levels for each gas.

The 120 ppb of O_3_ treatment used in our experiments might seem high, however, the exposure period was short (just five days). The relevance of using these amounts is the high O_3_ episodes which are not uncommon for example in southern Europe.

See S1-S3 Tables in S1 File for detailed information on the sequence of fumigations and the concentration of the gases in chamber 1.

### Greenhouse set up

After fumigation, the 32 plants per cohort (eight control, eight O_3_, eight CO_2_, eight O_3_ + CO_2_) were placed in the greenhouse in a completely randomized block design pattern. For the next five days after fumigation treatments, bees were introduced into the greenhouse and allowed to visit the EFNs.

### VOC sample collection and TD-GC-MS analysis

The volatile organic compounds (VOCs) emitted from EFNs were collected between 22^nd^ February and 8^th^ March 2020 between 09h00 and 16h00 when bees were most active. Quartz glass tubes (15 mm × 1.9 mm internal diameter) filled with tenax (1.5 mg) and carbotrap (1.5 mg) adsorbents were used to collect the VOCs. Both sides were closed with glass wool to keep the adsorption material in place (Jürgens et al 2006). Tubes were conditioned by heating them up for 30 min at 250 °C. EFNs on the upper three nodes (~12 EFNs) per plant were enclosed in polyacetate (oven) bags (Toppits, Minden, Germany) three hours prior to volatile collection. Subsequently, the air, containing the odors, was sucked trough the quartz glass tube for 15 minutes (1.1 l/min) using a battery-powered DC pump (Fürgut, Tannheim, Germany. The quartz glass tube was transferred into a glass-wool-packed thermodesorption tube and placed in the thermodesorper unit (TDU; TD100-xr, Markes, Offenbach am Main, Germany) connected to a GC/MS (Agilent 7890B GC and 5977 MS, Agilent Technologies, Palo Alto, USA). The thermodesorption tube, was heated up to 260 °C for 10 min. The desorbed components were transferred to the cold trap of the TDU (5 °C) to focus the analytes using N_2_ as carrier gas. The cold trap was heated up to 310 °C at a rate of 60 °C/sec and held for 5 min. The VOCs were transferred to the injector port of the GC/MS via a heated transfer line (300 °C) and with N_2_ as carrier gas. The GC was equipped with an HP-5MS UI capillary column (30 m × 0.25 mm × 0.25 μm, J&W Scientific, Folsom, CA, USA). Helium was used as carrier gas with constant pressure of 1 bar. The initial GC oven temperature was 40 °C for 1 min, then raised to 300 °C at 5 °C min^-1^ where it was held for 3 minutes. The transfer line temperature between GC and MS was 300 °C. The mass spectrometer was operated in electron impact (EI; 70 eV) ionization mode, scanning m/z from 40 to 650, at 2.4 scans s^-1^.

Volatile organic compounds (VOCs) were identified based on their mass spectra, which were compared with the MS Library (NIST 2.3) using Agilent MSD Productivity ChemStation (MSD ChemStation F.01.03.2357—Agilent Technologies, Inc.) software. Additionally, the identification was verified by the calculated Retention Indices (RI), based on our *n*-alkanes’ Retention Times (RT), and the values (RI) were compared with both the published values [48] and with the NIST Chemistry WebBook database. All potential contaminants (plastic softeners, column bleeding material etc.) and compounds with similar mass spectra were eliminated from the VOC table. We then compared each compound with similar mass spectra and retention index across all samples per treatment and only retained the compounds present in at least 50% of the samples per treatment (S4 Table in S1 File). We calculated the relative amount for each compound per sample in each treatment by taking the integrated peak area of each compound divided by the total area for all compounds. We used the relative ratio to perform NMDS and Random Forest analysis described in the data analysis section.

### Measurement of extra floral nectar volume and sugar concentration

Extra floral nectar was collected using graduated 5 μl micropipettes. Because the nectar volume from one EFN gland was relatively low, five random EFN glands per plant were used to estimate nectar volume. On each randomly selected EFN gland, extra floral nectar was tapped by placing one end of the micropipette to the EFN gland and the other end left open to allow the nectar to be drawn into the micropipette by capillary force. The micropipette was put in place until all the nectar had been drawn before moving to the next gland. Nectar volume was determined from the scale on the side of the micropipette.

Sugar concentration of nectar was measured using a portable optical light refractometer (Bellingham and Stanley–Eclipse 45–81 °Brix). The total nectar sugar concentration is expressed in °Brix, which is the sugar content of an aqueous solution. For the calculation of the quantity of sugar in the nectar, the optical light refractometers measure the percentage of sucrose on the Bx scale (1 °Bx is 1 g of sucrose in 100 g of solution). The total sugar concentration in the nectar is assessed by extracting the nectar from the flower with a micropipette and emptying the liquid onto the light refractometer’s glass prism. The ºBx is read by holding the device against light to determine its refractive index. The refractive index of nectar is used as a measure of sugar equivalent in the solution [49].

In total, we made three measurement rounds of nectar volume and sugar concentration per plant per treatment for each cohort.

### Bee visitation to extra floral nectaries

Observation of bee visits to EFN was done in two phases, each with 32 plants due to the greenhouse’s limited space. The first set of 32 plants were placed in the greenhouse for five continuous days from 23^rd^ to 27^th^ February 2020 and the second set from 3^rd^ to 7^th^ March 2020, each time with 50 individuals of *O*. *cornuta* obtained from Mauerbienen company (www.mauerbienen.com). Both sets were exposed to bees immediately after the end of the fumigation period. A group of four plants, one from each treatment, was observed for 15 continuous minutes twice a day (in the morning and afternoon) for five consecutive days. During this period, the number of bees visiting the plant’s extra floral nectaries was recorded.

### Data analysis

Statistical analyses were performed in R version 4.2.1 [50]. We used Non-metric Multidimensional Scaling (NMDS) to visualize the degree of chemical distances among VOCs based on their relative amounts in different treatments. To investigate which VOCs were associated with each treatment, we correlated values on the NMDS axes to metrics of VOCs based on Bray-Curtis dissimilarities with the vegan package version 2.6–2 in R. Adonis test, an implementation of PERMANOVA, was used to test for the significance of differences among the groups.

In the next step, we ran a random Forest model in MetaboAnalyst 5.0 (https://www.metaboanalyst.ca/) to determine which decisive compounds are most important for the separation of the groups [51]. First, we compared all VOCs between O_3_ and control treatments. Second, we compared these VOCs with O_3_ versus O_3_ + CO_2_. We did not have comparisons between CO_2_ with other treatments because this treatment was not significant in the NMDS analysis. We used the mean decrease of accuracy to interpret the VOCs importance in making the differences as suggested by [52]. According to [51], the mean decrease in accuracy (MDA) for a compound or variable is the normalized difference of the classification accuracy for the out-of-bag data when the data for that variable is included as observed, and the classification accuracy for the out-of-bag data when the values of the variable in the out-of-bag data have been randomly permuted.

We further constructed a second NMDS with only the decisive VOCs identified by the random Forest model to examine how they relate with the treatments, without all the other non-decisive chemicals tested in the first NMDS. Again, we used adonis test in PERMANOVA to test for significant differences among the groups.

To test for treatment effect on EFN nectar volume and sugar content, we used Linear Mixed Models (LMM) as implemented in the lme function (R package nlme). Here we used either nectar volume or sugar content as response variables and fitted each LMM with CO_2_ and O_3_ (and their interaction) as fixed effects, and plant ID as the random effect. We also constructed a LMM with bee visits as the response variable and used CO_2_, O_3_ (and their interaction), nectar volume, and sugar concentration as fixed factors to test whether these variables modulated the number of visits to EFNs. We retained the plant ID as the random effect. We used Tukey test to separate the means of treatments means where significant differences were found. Before analysis, each response variable was tested for normality using the Shapiro Wilk test [53]. If the test returned a significant result (p<0.05), the data were log-transformed to improve normality and fulfill the parametric tests’ assumptions before further analysis [54].

## Results

A bouquet of 101 VOC compounds (S4 Table in S1 File) was found in the headspace of *Vicia faba*. Out of these, 53 were identifiable. The VOC classes based on the number of compounds were alcohols (9%), Aldehydes (9%), Anisoles (2%), Benzoids (4%), Esters (5%) Ketones (7%), Terpenes (16%), and unknowns (48%)

### Effect of O_3_ and CO_2_ on VOCs

**Fig 2:**
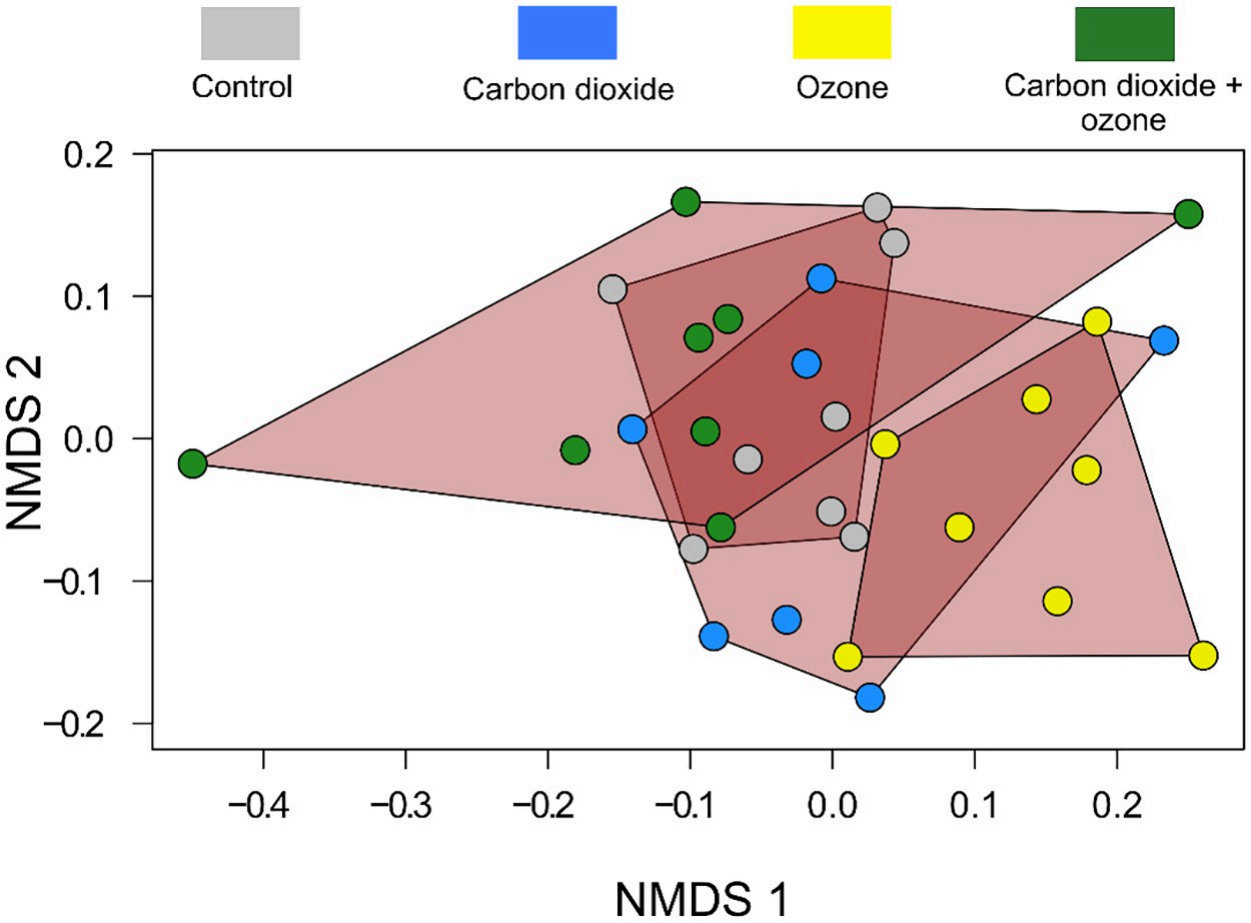
NMDS panel figure displaying the relationship between various Volatile Organic Compounds of *Vicia faba* plants’ headspace for each atmospheric treatment.

We found that both axes 1 and 2 of the first NMDS values could explain the variations in VOC values (Fig 2). Based on permutation test for adonis, the VOCs significantly differed between treatments (F_3,27_ = 2.193, P = 0.001). When further subjected to pairwise adonis comparisons, significant effects were observed due to differences between O_3_ versus control (adonis p = 0.006) and O_3_ versus O_3_ + CO_2_ (adonis p = 0.012), respectively.

**Fig 3:**
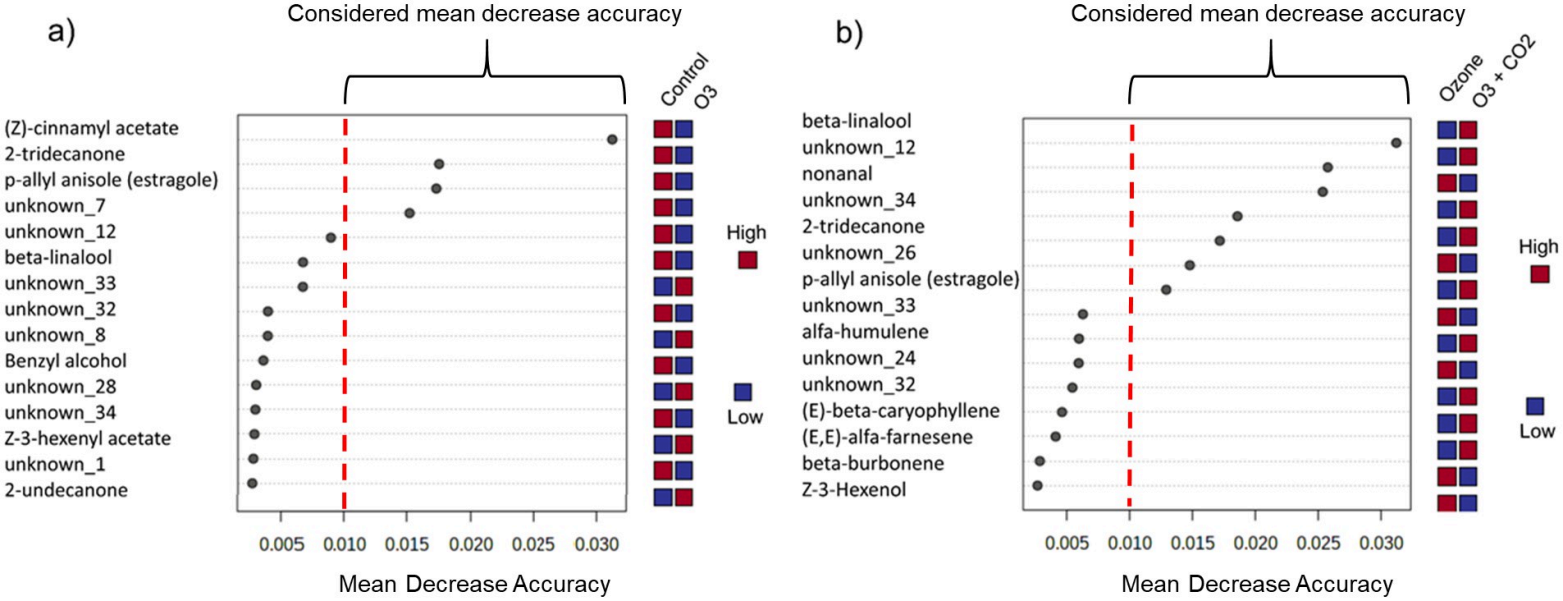
Outputs of the decisive VOCs identified by random Forest model relating to (a) control versus O_3_ and (b) O_3_ versus O_3_ + CO_2_. The compounds are ranked in decreasing order based on the model’s predictive accuracy from permuting the values in each feature.

Based on the random Forest model, we listed the top 15 decisive compounds for O_3_ versus control (Fig 3a) and O_3_ versus O_3_ + CO_2_ (Fig 3b) from the bouquet of 112 compounds based on their MDA. From the top 15 decisive compounds in each group, we identified four (Fig 3a) and seven compounds (Fig 3b), which had higher MDA value than the steepest point (MDA>0.010), as the most important ones for the separation of treatments. We therefore used the top four compounds in Fig 3a and top seven in Fig 3b for the second NMDS analysis described below.

**Fig 4:**
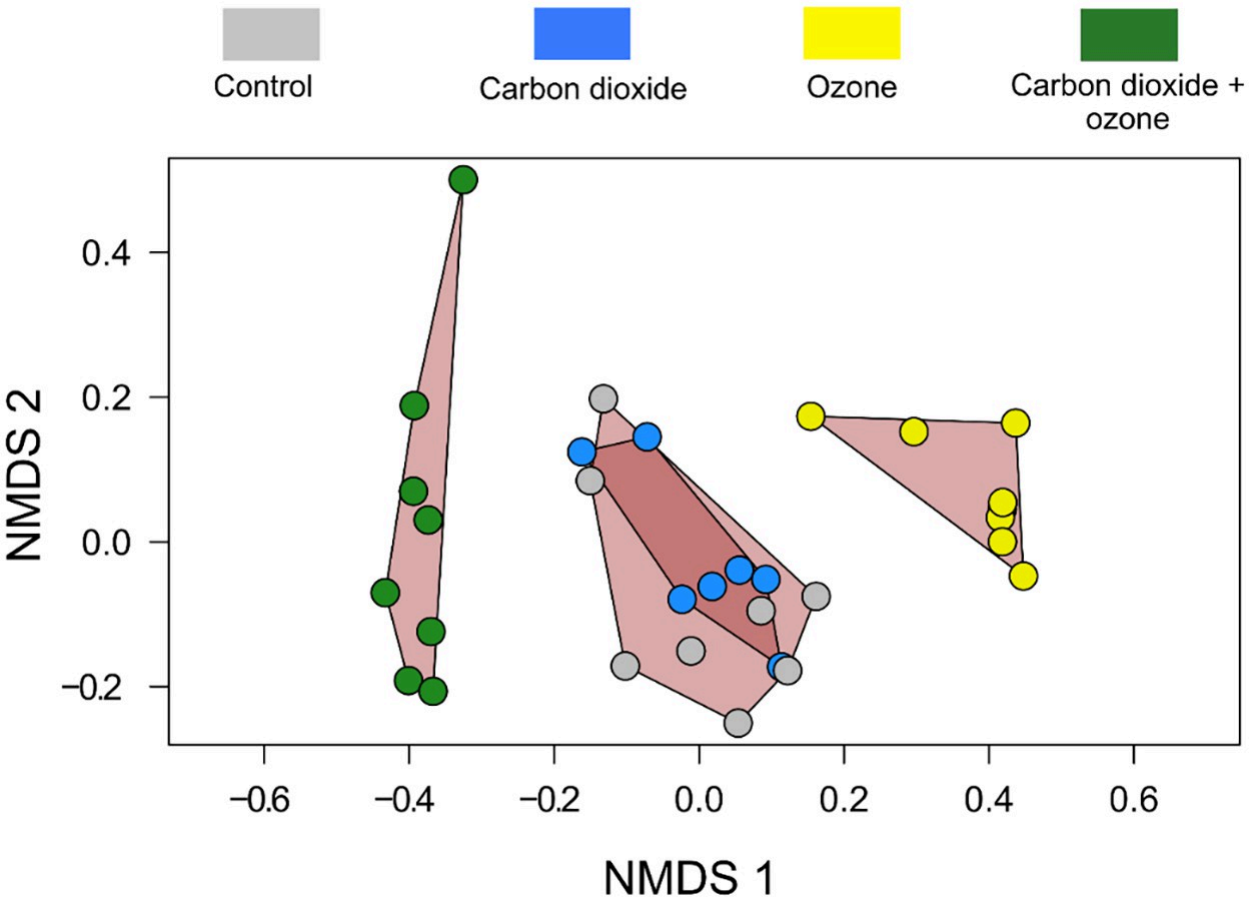
NMDS panel figure displaying the relationship between decisive Volatile Organic Compounds identified by the random Forest model and each atmospheric treatment.

In the second NMDS which was constructed with only the decisive VOCs identified by the random Forest model to relate these decisive VOCS with the treatments, both axes 1 and 2 of the NMDS values could explain the variations in the decisive VOC values (Fig 4). The decisive VOC composition significantly differed between treatments (F_1,29_ = 2.19, P<0.001). When further subjected to pairwise adonis comparisons, significant effects were observed due to differences between O_3_ versus control (adonis p = 0.003) and O_3_ versus O_3_ + CO_2_ (adonis p = 0.003), We also confirmed significant effects between control versus O_3_ + CO_2_ (adonis p = 0.009).

### Effects of O_3_ and CO_2_ on EFN nectar and consequences for bee visits

**Fig 5:**
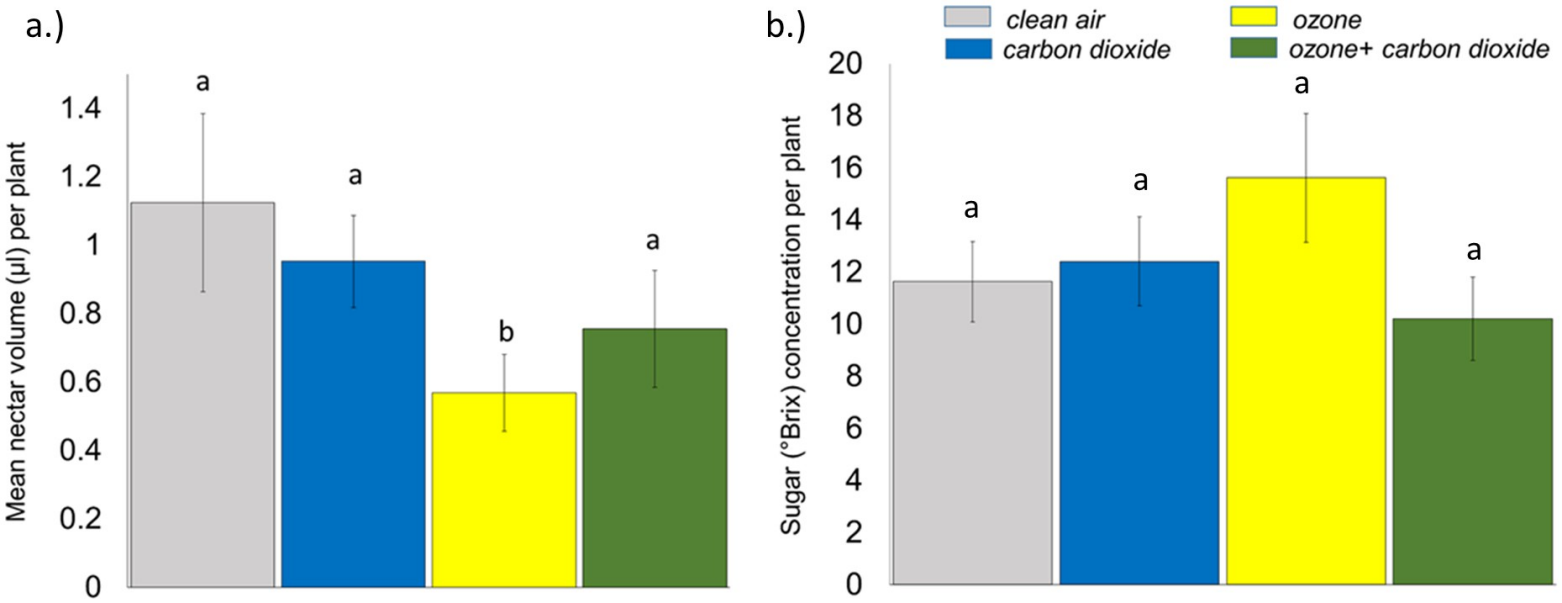
Volume (a) and sugar concentration (b) of nectar produced by five extra floral nectaries of plants in each atmospheric treatment. The different letters over the bars in the histogram indicate significant differences based LMMs. Same letters indicate no significant difference.

Plants from the control treatment produced the highest nectar volume (3.2 ± 0.8 μl), followed by the plants treated with elevated CO_2_ (2.8 ± 0.4 μl), a mixture of O_3_ and CO_2_ (2.2 ± 0.5 μl) and O_3_ (1.7 ± 0.3 μl), respectively (Fig 5a). For the nectar sugar concentration, the plants treated with O_3_ had 15.6 ± 2.5 °Bx, followed by CO_2_ (12.4 ± 1.7 °Bx), control treatment (11.6 ±1.5 °Bx), and a mixture of O_3_ and CO_2_ (10.2 ± 1.6 °Bx), respectively (Fig 5b).

Based on the LMMs, elevated O_3_ had a significant negative effect on nectar volume (t = -2.192, p = 0.032), while CO_2_ and the interaction between the two gases had no effect (p = 0.5 and p = 0.321), respectively (S5 Table in S1 File). For nectar sugar concentration, neither O_3_, CO_2_ nor their interaction had significant effects (p = 0.136, p = 0.768, and p = 0.102, respectively (Fig 5).

**Fig 6:**
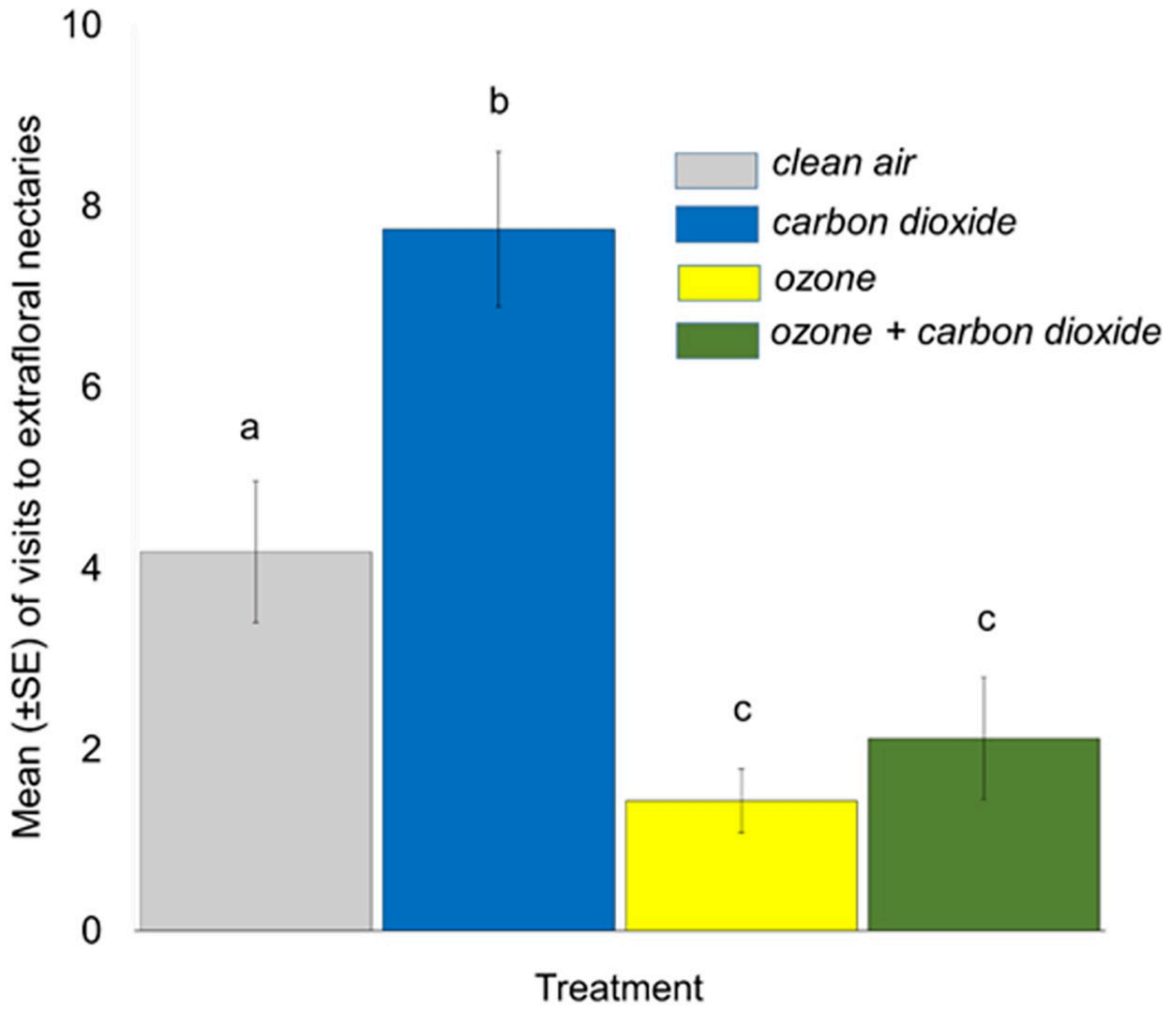
*Osmia cornuta* visits to EFNs on plants in each atmospheric treatment per plant over the ten-day sampling period. The different letters over the bars in the histogram indicate significant differences based on Tukey tests. Same letters indicate no significant difference.

A total of 246 bee visits to EFNs were recorded. Most visits were to plants exposed to elevated CO_2_ (Mean 7.8 ± 0.9 SE per plant within the ten days of sampling), followed by visits to control plants (4.2 ± 0.8), to a mixture of CO_2_ and O_3_ (2.1 ± 0.7), and to O_3_ (1.4 ± 0.4), respectively (Fig 6). CO_2_ enhanced bee visits (t = 3.105, p = 0.03) but O_3_ and O_3_ + CO_2_ had negative effects on bee visits (t = -2.229, p = 0.30 and t = -2.081, p = 0.042, respectively).

## Discussion

Our results show that increased O_3_ had significantly negative effects on the blends of VOCs emitted, nectar volume secreted by the EFNs, and bee visits. There is a possibility O_3_ affects these secondary-metabolites and bee visits through plant physiology [55]. CO_2_ combined with enhanced O_3_ levels showed distinct VOC profiles and significantly lower visitation rates compared to plants only exposed to enhanced CO_2_ concentrations. Our results underpin that the understanding of plant-mediated responses requires the consideration of interactive effects of different atmospheric compounds.

### Effect of O3 and CO2 on VOCs

We found that the VOC blend changed when the plants were treated with elevated O_3_. Because the VOCs were trapped after the plants’ exposure to O_3_ and or CO_2_, the results indicate effects mediated via modified plants physiology. Our study findings are consistent with other studies, notably: [56–59], where collection of VOCs was performed after exposure to O_3_ had ceased.

From our findings, it is highly likely that O_3_ indirectly affected the emission of VOCs through modified plant physiology. This modification could either be through decreased photosynthesis due to reduced stomatal conductance [8,9] or the effects of O_3_ on Rubisco [11], which have a net negative impact on biomass accumulation [8,9]. Our results indicate that O_3_-stressed plants with presumably reduced energy reserves, lower primary productivity and further changes in plant metabolism have a lower net emission of VOCs than a plant under normal environmental conditions [60].

### Effect of O_3_ on EFN nectar production

Elevated O_3_ had a significant negative effect on nectar volume, while CO_2_ had no effect. Little is known about how O_3_ influences changes in nectar secretion in Fabaceae, but it may influence resource allocation to vital plant parts, e.g., leaves, stems, flowers, storage organs, and extra floral nectaries [see 61]. These changes could have knock-on effects on nectar production [62].

When O_3_ is sequestered into plant tissue, e.g., the leaf parenchyma, it is partially broken down to derivatives that can affect photosynthesis [61]. This damage affects the demand for carbon needed to support the repair process, as the plant demands more carbohydrates [63]. Carbohydrates, primarily glucose, sucrose, and fructose, are the most abundant nutrients in nectar [64]. The phloem tissue is responsible for synthesizing these essential compounds of nectar [64]. In *Vicia faba* plants, the nectary tissue can also be photosynthetically active since it contains chloroplasts in the nectary parenchyma [65]. O_3_ can influence many factors identified contributing to the phloem constituents, the chloroplasts in the nectary tissue, and the amount of nectar produced by these plant parts [see 66,67]. It is also possible that elevated ozone affected water availability/water allocation reducing the volume of EFN. This is because in response to elevated ozone, plants close their stomata which affect their transpiration rate [8,9]. These effects would have explained our results for nectar volume production under the O_3_ treatment.

Nectar sugar concentration was not significantly influenced by O_3_, although there was a positive trend for plants exposed to O_3_ compared to other treatments. Previous studies have found that the effects of O_3_ on monosaccharides and total soluble carbohydrates, including total sugars, depend on the severity of the stress [55], which is a function of dosage and the length of exposure [3]. It is noteworthy that the Nectar sugar concentration is higher in O_3_ exposed plants, which have low nectar volume.

### Behavioral consequences of increased O_3_ and CO_2_ on bees utilizing EFNs

*Osmia cornuta* bee visits to EFNs were significantly higher on plants exposed to elevated CO_2_ but significantly lower on plants exposed to O_3_ and a mixture of O_3_ and CO_2_. From our results, bees had a stronger preference for CO_2_-treated plants than we would assume from the nectar volume as the mean was higher for control than CO_2_ treated plants, while visitation rates was two-fold higher for CO_2_ plants. This might indicate that further changes in the VOC profiles/blend are added to the attractiveness of the EFNs in this treatment. We can more generally draw the conclusion from these results that, in addition to the effects mediated by plant nectar volume and sugar concentration, changes in the relative amounts of VOCs in the different treatments may also explain the high or low visitation rates. It is well established that plants attract and maintain their appeal to pollinators primarily via visual cues and VOCs, which enables pollinators to find appropriate flowers to collect nectar and pollen [68]. It may be possible that ozone alters color of flowers and leaves via oxidative stress [69]. However, we did not observe a change in the color of flowers under O_3_ stress but injuries on leaves. The VOCs play an essential role in the discernment of the level and composition of resources, enabling insects to forage on the most rewarding plants [70,71]. O_3_, however, is reported to affect this plant-pollinator communication by altering the VOC blend making it difficult for pollinators to find appropriate flowers [see 13]. A recent review paper by [55] reports that elevated O_3_ alters the composition and diversity of plant communities through its effects on key physiological functions, and changes the emission of VOCs, which affect plant-insect interactions and the composition of insect communities.

It should be noted that nectar volume is a secondary factor affected by O_3_ to regulate bee visits. In general, nectar volume functions as an attractive factor in bee visits and O_3_ makes nectar volume significantly lower than control. Some of the VOCs such estragole, and beta-linalool that we identified are the most dominant VOCs emitted by the flowers of a variety of plant species, especially those attractive to pollinators [72–74]. These VOCs were amongst the most severely affected VOCs by O_3_ in our study. Estragole is an important insect pollinator attractant for plants such as oil palm [74]. Often pollinators react to specific ratios of volatiles in a blend, rather than to specific volatiles. Our results indicate that O_3_ altered some ratios of VOC composition that are important for pollinator recognition.

In our study, we found significant negative effects of O_3_ on bee visitation, nectar volume, and VOCs, which may have provided a different olfactory cues to the bees compared to the CO_2_ treated plants, making it less apparent to find the most resourceful plants [75]. Previous findings have found correlations between the frequency of visits by *Osmia* bees and the degree of rewarding (e.g., nectar volume) [76]. For instance, female Osmia bees were found to use nectar VOCs to identify the most rewarding *Penstemon caesius* (Plantaginaceae) flowers before landing on the corolla [76]. *Osmia* species have innate preferences towards specific blends of VOCs typical of their host plants [77,78]. Therefore, they may easily find their appropriate plants, instead of plants that do not fit their inner reference templates (e.g. O_3_-treated plants).

The exposure of plants to CO_2_ is likely to have increased the attractiveness of the plants to bee visitors through increased biomass accumulation due to increased photosynthesis. Although we did not measure photosynthetic activity, future investigations should determine if physiological changes in plants due to CO_2_ exposure can cause enhanced attractiveness. Previous studies have established that *Vicia faba* invests in extra assimilate in the initiation and maintenance of flowers at elevated CO_2_, increasing overall floral display [see 1]. An increase in floral display or longevity may be due to the changes in resource allocation within the plant. From an energy efficiency perspective, bees generally have more visits to plants with more flowers (and EFNs) and higher floral display because they can utilize less energy to forage on more flowers in a small area [79]. The EFNs on the plants with higher floral display stand a better chance of being visited, which is the likely case in our study.

## Conclusion

Our results showed that elevated O_3_ levels altered the VOC composition and the amount of EFN nectar reward, threatening the interactions between bee pollinators and *Vicia faba* plants, as shown to reduce bee visits in this research. While, within the scope of this study, increases in O_3_ level were associated with changes in VOCs relevant to bee attraction to the flowers, nectar volume, and bee visitation, CO_2_ seemed to partly but not significantly compensate for these effects. Our results add essential information to the general knowledge on the effects of O_3_ and CO_2_ on the profiles of volatiles emitted by *Vicia faba* plants and bee responses to these changes. Further work is needed to test the responses of bees to the individual VOCs and different blends if the composition and relative amounts of different VOCs play an important role, to determine the thresholds at which they respond to these compounds to fully understand the behavioural responses of different pollinator species to plants exposed to greenhouse gases.

As greenhouse gas levels continue to rise globally, it is important to take these findings interactive effects with enhanced O_3_ levels into serious consideration to better prepare for future scenarios of plant responses and threats for plant-insect interactions.

## Supporting information

S1 File (PDF)
