# Supplementary material for: Elevated ozone and carbon dioxide affects the composition of volatile organic compounds emitted by Vicia faba (L.) and visitation by European orchard bee (Osmia cornuta)
Source: PLoS One. 2023 Apr 26;18(4):e0283480. doi: 10.1371/journal.pone.0283480 (PMC10132541; doi:10.1371/journal.pone.0283480)
Supplement: S1 File — (PDF) [file pone.0283480.s001.pdf]

Supporting information

S1 Table. Sequence of fumigation of a cohort of field bean plants with ozone and carbon dioxide over a period of five days.

Day	Treatment	Chamber No.	Time*	Remarks
1	Carbon dioxide + ozone	1	5 am – 10 am	Ran concurrently
	Control	2	5 am – 10 am	
	Ozone	1	10 am – 3 pm	
	Carbon dioxide	1	3 pm – 8 pm	
2	Ozone	1	5 am – 10 am	
	Carbon dioxide	1	10 am – 3 pm	Ran concurrently
	Control	2	10 am – 3 pm	
	Carbon dioxide + ozone	1	3 pm – 8 pm	
3	Carbon dioxide	1	5 am – 10 am	
	Carbon dioxide + ozone	1	10 am – 3 pm	
	Control	2	3 pm – 8 pm	Ran concurrently
	Ozone	1	3 pm – 8 pm	
4	Carbon dioxide + ozone	1	5 am – 10 am	Ran concurrently
	Control	2	5 am – 10 am	
	Ozone	1	10 am – 3 pm	
	Carbon dioxide	1	3 pm – 8 pm	
5	Ozone	1	5 am – 10 am	
	Control	2	10 am – 3 pm	Ran concurrently
	Carbon dioxide	1	10 am – 3 pm	
	Carbon dioxide + ozone	1	3 pm – 8 pm	

***Note**: The fumigation start and end times indicated have been rounded to the nearest hour. The placement of a new set of plants in the chambers after the previous set was fumigated took some time (~5-10 minutes), but each fumigation cycle always lasted five continuous hours. The time interval between the fumigation of one set of plants with one gas to the next was sufficient for the gas levels inside the chamber to drop to 0 ppb for ozone and ~400 for carbon dioxide once the generator/inlets were switched off, and the chamber's door was open. Once fumigation began, gas levels increased rapidly to the required amount within ~5 – 7 minutes.*

S2 Table. Mean carbon dioxide (ppm) and ozone (ppb) concentration during fumigation in each treatment.

Treatment	Gas in chamber	Mean	Standard deviation
Carbon dioxide only	Carbon dioxide	868	141
	Ozone	1	0
Carbon dioxide + ozone	Carbon dioxide	842	210
	Ozone	115	9
Ozone only	Carbon dioxide	346	122
	Ozone	117	8

S3 Table. Mean logger data readings for carbon dioxide and ozone levels in the fumigation chambers per treatment in each of the two cohorts.

Cohort	Treatment	Gas measured	Date	Time	Mean reading during	SD
Osmia cohort 1**	Carbon dioxide + ozone	CO2	17-Feb-20	5 am -10 am	694.722	290.989
	Carbon dioxide only	CO2	17-Feb-20	10 am - 3 pm	1007.399	382.593
	Ozone only	CO2	17-Feb-20	3 pm - 8 pm	665.750	355.359
	Ozone only	CO2	18-Feb-20	5 am -10 am	478.379	307.135
	Carbon dioxide + ozone	CO2	18-Feb-20	10 am - 3 pm	1078.704	218.551
	Carbon dioxide only	CO2	18-Feb-20	3 pm - 8 pm	424.785	185.639
	Ozone only	CO2	19-Feb-20	5 am -10 am	348.657	30.119
	Carbon dioxide only	CO2	19-Feb-20	10 am - 3 pm	1083.170	183.899
	Carbon dioxide + ozone	CO2	19-Feb-20	3 pm - 8 pm	1093.616	174.927
	Carbon dioxide + ozone	CO2	20-Feb-20	5 am -10 am	483.015	359.073
	Ozone only	CO2	20-Feb-20	10 am - 3 pm	345.885	140.781
	Carbon dioxide only	CO2	20-Feb-20	3 pm - 8 pm	1021.253	164.264
	Carbon dioxide only	CO2	21-Feb-20	5 am -10 am	423.214	283.522
	Carbon dioxide + ozone	CO2	21-Feb-20	10 am - 3 pm	1107.702	219.308
	Ozone only	CO2	21-Feb-20	3 pm - 8 pm	371.252	148.678
Osmia cohort 2**	Carbon dioxide + ozone	CO2	26-Feb-20	5 am -10 am	783.201	445.563
	Carbon dioxide only	CO2	26-Feb-20	10 am - 3 pm	1312.636	242.557
	Ozone only	CO2	26-Feb-20	3 pm - 8 pm	567.185	249.496
	Ozone only	CO2	27-Feb-20	5 am -10 am	461.934	18.930
	Carbon dioxide only	CO2	27-Feb-20	10 am - 3 pm	1271.884	163.955
	Carbon dioxide + ozone	CO2	27-Feb-20	3 pm - 8 pm	505.285	163.114
	Carbon dioxide + ozone	CO2	28-Feb-20	5 am -10 am	955.516	568.622
	Ozone only	CO2	28-Feb-20	10 am - 3 pm	493.102	100.965
	Carbon dioxide only	CO2	28-Feb-20	3 pm - 8 pm	1284.133	103.364
	Carbon dioxide only	CO2	29-Feb-20	5 am -10 am	618.855	418.033
	Carbon dioxide + ozone	CO2	29-Feb-20	10 am - 3 pm	1268.335	191.363
	Ozone only	CO2	29-Feb-20	3 pm - 8 pm	455.743	65.840
	Ozone only	CO2	01-Mar-20	5 am -10 am	420.326	20.212
	Carbon dioxide + ozone	CO2	01-Mar-20	10 am - 3 pm	1275.823	182.278
	Carbon dioxide only	CO2	01-Mar-20	3 pm - 8 pm	1269.402	32.767

** denotes Ozone graph produced instead of values; ** denotes Ozone logger battery ran out*

S4 Table: Comparison of the chemical composition of volatile organic compounds found in *Vicia faba* plants' headspace by TDU-GC-MS across all treatments. The values show means ± SE.

Chemical Class	Chemical name	Calculated Retention Index	Control	Carbon dioxide	Ozone	Ozone +carbon dioxide
Unknown	unknown_1	670	0.168±0.038	0.081±0.037	0.077±0.035	0.064±0.021
Unknown	unknown_2	676	0.213±0.052	0.15±0.045	0.19±0.043	0.134±0.023
Unknown	unknown_3	688	0.466±0.114	0.556±0.099	0.444±0.056	0.355±0.129
Ketone	acetoin	709	0.475±0.113	0.268±0.053	0.347±0.043	0.198±0.05
Unknown	unknown_4	713	0.318±0.093	0.34±0.081	0.375±0.066	0.355±0.128
Unknown	unknown_5	738	0.109±0.027	0.108±0.03	0.177±0.025	0.15±0.021
Unknown	unknown_6	754	0.647±0.244	1.003±0.198	1.154±0.168	1.101±0.333
Aromatic hydrocarbons	toluene	764	0.477±0.08	0.497±0.068	0.466±0.045	0.451±0.1
Aldehyde	3-methyl-2-butenal	786	0.477±0.062	0.22±0.08	0.527±0.1	0.292±0.106
Unknown	unknown_7	792	0.838±0.22	0.668±0.306	0±0	0.094±0.084
Unknown	unknown_8	795	0.165±0.061	0.336±0.092	0.259±0.023	0.31±0.051
Aldehyde	hexanal	802	1.393±0.387	1.125±0.17	1.249±0.173	0.981±0.191
Unknown	unknown_9	815	0.133±0.034	0.158±0.038	0.199±0.024	0.172±0.034
Unknown	unknown_10	840	0.129±0.017	0.08±0.024	0.106±0.019	0.095±0.021
Alcohol	Z-3-hexenol	855	2.098±0.633	2.562±1.455	7.748±2.054	1.882±0.964
Unknown	unknown_11	887	0.82±0.141	0.64±0.051	0.893±0.122	0.844±0.14
Terpene	styrene	891	1.262±0.448	1.454±0.267	1.103±0.219	1.242±0.309
Aldehyde	heptanal	903	0.762±0.157	0.764±0.099	1.2±0.258	0.719±0.147
Unknown	unknown_12	908	0.654±0.223	0.92±0.206	0±0	0.866±0.23
Ketone	2-hydroxy-2-cyclopenten-1-one	926	0.797±0.143	0.683±0.111	0.593±0.068	0.667±0.129
Terpene	alfa-pinene	933	0.105±0.019	0.108±0.026	0.112±0.02	0.169±0.045
Unknown	unknown_13	943	0.146±0.031	0.172±0.025	0.186±0.025	0.168±0.035
Unknown	unknown_14	957	0.239±0.078	0.137±0.071	0.375±0.132	0.176±0.043
Aldehyde	benzaldehyde	961	1.333±0.169	1.319±0.156	1.351±0.053	1.152±0.186
Unknown	unknown_15	968	0.184±0.02	0.178±0.023	0.224±0.014	0.196±0.041
Unknown	unknown_16	972	0.114±0.034	0.131±0.023	0.041±0.041	0.073±0.033
Unknown	unknown_17	981	1.325±0.384	0.27±0.27	1.703±0.368	1.649±0.292
Unknown	unknown_18	987	1.165±0.389	0.198±0.198	0.353±0.231	0.509±0.228
Ketone	sulcatone	989	1.559±0.285	1.46±0.243	1.8±0.347	1.447±0.277
Unknown	unknown_19	993	1.416±0.328	0.706±0.309	1.496±0.175	0.986±0.321
Ester	Z-3-hexenyl acetate	1010	6.095±0.816	8.179±3.619	11.058±4.357	6.887±3.772
Ester	hexyl acetate	1016	0.179±0.044	0.224±0.126	0.425±0.15	0.195±0.153
Unknown	unknown_20	1019	0.273±0.062	0.212±0.04	0.353±0.082	0.363±0.079
Unknown	unknown_21	1023	0.15±0.029	0.238±0.058	0.244±0.026	0.107±0.035
Terpene	p-cymene	1025	0.229±0.04	0.374±0.087	0.3±0.025	0.265±0.04
Terpene	D-limonene	1029	1.425±0.462	1.098±0.265	1.369±0.635	2.523±0.919
Alcohol	2-ethyl-1-hexanol	1031	1.47±0.678	1.147±0.118	1.261±0.312	1.074±0.41
Alcohol	benzyl alcohol	1036	0.302±0.145	0.198±0.052	0.048±0.033	0±0
Terpene	Z-beta-ocimene	1040	0.56±0.092	0.424±0.082	0.392±0.082	0.746±0.379
Unknown	unknown_22	1042	0.214±0.041	0.217±0.053	0.269±0.023	0.19±0.024
Aldehyde	phenylacetaldehyde	1045	0.412±0.044	0.45±0.051	0.37±0.073	0.291±0.031

Terpene	E-beta-ocimene	1050	3.782±0.905	3.721±1.235	2.774±0.878	2.609±0.627
Unknown	unknown_23	1055	0.55±0.085	0.554±0.103	0.74±0.062	0.573±0.071
Unknown	unknown_24	1059	0.486±0.104	0.386±0.099	0.584±0.062	0.319±0.032
Unknown	unknown_25	1062	0.159±0.049	0.178±0.087	0.108±0.032	0.071±0.03
Unknown	unknown_26	1064	0.216±0.073	0.219±0.075	0.431±0.055	0.174±0.028
Ketone	acetophenone	1068	1.007±0.119	0.971±0.194	0.914±0.144	0.643±0.074
Unknown	unknown_27	1071	0.338±0.05	0.409±0.054	0.431±0.024	0.349±0.04
Alcohol	octanol	1073	0.493±0.061	0.435±0.068	0.73±0.129	0.421±0.072
Unknown	unknown_28	1091	0.314±0.052	0.452±0.049	0.485±0.044	0.362±0.037
Unknown	unknown_29	1094	1.083±0.361	0.94±0.222	0.938±0.243	1.013±0.117
Terpene	beta-linalool	1101	0.778±0.258	0.99±0.234	0±0	5.562±2.59
Aldehyde	nonanal	1106	2.84±0.365	2.762±0.216	3.084±0.279	0.186±0.186
Unknown	unknown_30	1109	0.222±0.08	0.185±0.108	0.308±0.12	0.08±0.032
Unknown	unknown_31	1112	0.402±0.047	0.393±0.066	0.457±0.068	0.385±0.081
Unknown	unknown_32	1115	0.355±0.097	0.074±0.074	0.063±0.063	0.299±0.053
Unknown	unknown_33	1117	0.555±0.137	0.942±0.129	1.113±0.13	0.512±0.113
Terpene	p-menthatriene	1124	0.526±0.14	0.298±0.118	0.339±0.113	0.208±0.158
Terpene	cosmene	1132	1.065±0.251	1.074±0.127	1.05±0.263	1.355±0.599
Terpene	alpha-ocimene	1145	0.645±0.112	0.58±0.213	0.564±0.146	0.417±0.1
Unknown	unknown_34	1165	0.175±0.058	0±0	0.015±0.015	0.202±0.029
Unknown	unknown_35	1172	1.11±0.05	1.194±0.144	1.208±0.142	0.972±0.161
Unknown	unknown_36	1174	0.903±0.22	0.732±0.099	0.991±0.321	0.887±0.105
Polycyclic aromatic hydrocarbons	naphthalene	1183	0.37±0.098	0.454±0.093	0.852±0.331	0.704±0.41
Unknown	unknown_37	1186	0.31±0.073	0.281±0.12	0.288±0.097	0.378±0.056
Ester	E-3-hexenyl butyrate	1189	0.194±0.062	0.245±0.063	0.193±0.065	0.048±0.048
Unknown	unknown_38	1193	0.486±0.086	0.59±0.113	0.558±0.112	0.508±0.216
Ester	methyl salicylate	1196	0.973±0.261	0.505±0.19	0.539±0.204	0.592±0.179
Anisole	estragole	1201	2.379±0.748	2.536±0.799	0.4±0.4	4.024±1.105
Aldehyde	decanal	1208	4.649±0.381	4.95±0.646	5.182±0.263	4.761±0.579
Unknown	unknown_39	1216	0.33±0.054	0.519±0.069	0.494±0.071	0.349±0.048
Aldehyde	Z-cinnamaldehyde	1222	1.888±1.28	0.559±0.116	0.522±0.143	0.655±0.115
Unknown	unknown_40	1225	0.609±0.098	0.746±0.134	0.751±0.126	0.517±0.048
Unknown	unknown_41	1230	0.273±0.072	0.446±0.109	0.477±0.134	0.27±0.054
Alcohol	3-phenylpropanol	1233	0.622±0.077	0.992±0.453	0.278±0.101	0.643±0.157
Unknown	unknown_42	1236	0.304±0.088	0.225±0.121	0.234±0.074	0.106±0.033
Unknown	unknown_43	1261	0.202±0.06	0.418±0.066	0.414±0.101	0.189±0.046
Alcohol	Z-cinnamyl alcohol	1264	1.158±0.225	1.072±0.461	0.615±0.121	0.805±0.163
Aldehyde	E-cinnamaldehyde	1273	0.971±0.395	0.719±0.486	0±0	0.993±0.517
Unknown	unknown_44	1280	1.397±0.991	0.429±0.161	0.423±0.073	1.175±0.824
Unknown	unknown_45	1282	0.903±0.157	1.386±0.265	2.99±1.521	0.992±0.187
Anisole	E-anethole	1288	0.269±0.137	0.488±0.108	0.189±0.065	0.25±0.081
Polycyclic aromatic hydrocarbons	1-methyl-naphtalene	1294	0.39±0.025	0.247±0.131	0.333±0.132	0.298±0.064
Ketone	2-undecanone	1296	0.85±0.044	1.039±0.153	0.856±0.117	0.887±0.096
Alcohol	E-cinnamyl alcohol	1309	2.253±0.492	1.35±0.996	1.527±0.177	2.859±1.143

Unknown	unknown_46	1346	1.276±0.343	1.272±0.41	1.168±0.318	0.565±0.141
Unknown	unknown_47	1359	0.157±0.045	0.123±0.11	0.132±0.062	0.148±0.082
Ketone	gamma-nonalactone	1366	1.023±0.144	1.102±0.199	1.119±0.155	0.796±0.134
Terpene	alfa-copaene	1380	0.691±0.132	0.023±0.023	0.374±0.176	0.203±0.135
Terpene	beta-burbonene	1388	0.43±0.096	0.564±0.115	0.568±0.137	0.32±0.055
Ester	Z-cinnamyl acetate	1393	0.779±0.076	0.305±0.153	0±0	0.285±0.196
Terpene	beta-elemene	1395	0.244±0.091	0.392±0.215	0.129±0.098	0.285±0.103
Polycyclic aromatic hydrocarbons	2,6-dimethyl naphtalene	1404	0.39±0.106	0.419±0.199	0.327±0.142	0.281±0.079
Alcohol	O-methyl eugenol	1408	0.362±0.179	0.249±0.167	0.113±0.113	0.713±0.301
Unknown	unknown_48	1420	0.472±0.083	0.555±0.182	0.423±0.145	0.272±0.076
Terpene	E-beta-caryophyllene	1424	8.951±1.961	9.548±2.273	5.121±0.956	11.845±2.298
Ketone	Z-geranylacetone	1456	1.108±0.362	1.74±0.695	1.556±0.203	1.094±0.168
Terpene	alfa-humulene	1458	1.256±0.308	1.515±0.379	0.703±0.181	1.483±0.191
Alcohol	1-dodecanol	1476	2.526±0.238	2.595±0.165	2.846±0.269	2.373±0.295
Ketone	2-tridecanone	1498	1.064±0.172	1.803±0.295	0.27±0.27	1.083±0.102
Terpene	E,E-alfa-farnesene	1512	1.261±0.46	0.955±0.43	0.5±0.334	1.789±0.519

S5 Table. LMM outputs of nectar volume, amount of sugar, and bee visitation to extra floral nectaries in field bean plants under different treatments.

Response variable	Fixed factor(s)	Estimate	Std. Error	t-value	p-value
Nectar volume	Carbon dioxide	-0.172	0.253	-0.678	0.500
	**Ozone**	**-0.555**	**0.253**	**-2.192**	**0.032**
	Carbon dioxide: Ozone	0.358	0.358	1.000	0.321
Nectar sugar concentration	Carbon dioxide	0.781	2.641	0.296	0.768
	Ozone	3.990	2.641	1.511	0.136
	Carbon dioxide: Ozone	-6.198	3.734	-1.660	0.102
Bee visits	**Carbon dioxide**	**0.299**	**0.096**	**3.105**	**0.003**
	**Ozone**	**-0.233**	**0.105**	**-2.229**	**0.030**
	**Nectar volume**	**0.124**	**0.054**	**2.306**	**0.025**
	Sugar concentration	-0.001	0.005	-0.276	0.784
	**Carbon dioxide: Ozone**	**-0.296**	**0.142**	**-2.081**	**0.042**

Note: *Figures in* ***bold*** *denote significant results.*
